# Estradiol induces transcriptional and posttranscriptional modifications in versican expression in the mouse uterus

**DOI:** 10.1007/s10735-012-9476-1

**Published:** 2012-12-28

**Authors:** Renato M. Salgado, Ambart C. Covarrubias, Rodolfo R. Favaro, Caroline Serrano-Nascimento, Maria Tereza Nunes, Telma M. T. Zorn

**Affiliations:** 1 Department of Cell and Developmental Biology, University of São Paulo, Avenida Professor Lineu Prestes, 1524—Room 429, Butantã, São Paulo, SP 05508-000 Brazil; 2 Department of Physiology and Biophysics, Institute of Biomedical Sciences, University of São Paulo, São Paulo, Brazil

**Keywords:** Estradiol, Versican, mRNA stability, Mouse uterus, Poly(A) tail

## Abstract

We have previously shown the differential expression of versican in the mouse uterus under ovarian hormone influence. We also demonstrated there is not a direct correlation between mRNA levels and protein expression, suggesting posttranscriptional events, such as alteration in mRNA stability. This posttranscriptional effect may result in the elongation and stabilization of transcripts poly(A) tail. Thus, the aim of this study was to analyze whether estradiol (E2) regulates versican mRNA stability and expression in a dose-related and time-dependent manner. For this purpose female mice were ovariectomized and treated with a single injection of 0.1 or 10 μg E2. To block transcription a group of females received a single injection of alpha-amanitin before hormone administration. Uterine tissues were collected 30 min, 1, 3, 6, 12 and 24 h after treatments and processed for quantitative real time PCR (qPCR), RACE-PAT Assay and immunohistochemistry. qPCR showed that versican mRNA levels are higher than control from 3 to 24 h after E2 administration, whereas after transcription inhibition versican mRNA unexpectedly increases within 3 h, which can be explained when transcriptional blockers alter the degradation rate of the transcript, resulting in the superinduction of this mRNA. Accordingly, analysis of versican transcript poly(A) tail evidenced a longer product 3 h after treatment, but not after 12 h. Versican immunoreaction becomes conspicuous in the superficial stroma only 3 h after E2 injection, whereas the whole stroma is immunoreactive from 6 h onward. These results demonstrate that E2 modulates versican at the transcriptional and posttranscriptional levels in a time-dependent manner.

## Introduction

The extracellular matrix (ECM) is a complex structure of macromolecules capable of self-assembly and is composed predominantly of collagens, non-collagenous multiadhesive glycoproteins, elastin, hyaluronan and proteoglycans (Kresse and Schönherr 2001). The ECM of the uterine tissues plays important functions in endometrial decidualization, embryo implantation, trophoblast cell invasion and the maintenance of gestation (Aplin et al. 1988; Iwahashi et al. 1996; Abrahamsohn and Zorn 1993; Aplin 2002). Data from our group have demonstrated that in mice, the deposition of collagens and proteoglycans in the uterine tissue varies along the estrous cycle and during early pregnancy (San Martin et al. 2003; Spiess et al. 2007; Salgado et al. 2009a). In addition, using a model of ovariectomy and hormone replacement as experimental strategy, it was demonstrated that the expression and deposition of proteoglycans, such as the small leucine-rich proteglicans biglycan, decorin, fibromodulin and lumican, as well as versican, are differentially modulated in the uterine tissues by estradiol (E2) and progesterone (P4) (Salgado et al. 2009b, 2011).

Versican is a large chondroitin sulfate proteoglycan that belongs to the family of hyaluronan-binding proteoglycans termed hyalectins, and is found in many soft tissues. It influences the formation of the ECM network and modulates cell–matrix and cell–cell interactions, acting on cell proliferation and differentiation (Shinomura et al. 1995; Zimmermann 2000; Wight 2002). A previous study (Salgado et al. 2009b) has shown that the expression and distribution of versican in the mouse uterus are region-specifically modulated by ovarian hormones E2 and P4. This work also demonstrated that there is not always a direct correlation between mRNA levels and protein deposition, indicating that posttranscriptional events, such as regulation of RNA stability, may be involved in this modulation process.

The biological effects of E2 and P4 are mediated by nuclear and membrane-associated receptors. At the nucleus, these receptors act as transcription factors regulating gene expression by direct binding to DNA regulatory sequences of genes and by interactions with other transcription factors, such as c-fos and c-jun, in a process modulated by co-regulators (Lydon et al. 1995; Weihua et al. 2000).

Membrane-associated steroid receptors of rapid action have been described, and their effects are mediated by protein G-associated mechanisms (Song et al. 2005). Alteration in mRNA stability may be induced by non-genomic action of steroid hormones (Review in Ing 2005). One of these posttranscriptional effects is the elongation of mRNA poly(A) tail, making transcripts more stable and efficiently translated, thus generating more protein. For instance, the ECM glycoprotein fibulin-1 is differentially modulated in an ovarian cancer cell line. The expression of both splice variants, fibulin-1C and fibulin-1D, is influenced by E2 at the transcriptional level. However, only fibulin-1D mRNA is posttranscriptionally regulated, by reduction of its half-life from 8 to 5.5 h (Bardin et al. 2005).

Several studies demonstrate that E2 regulates ECM remodeling, through synthesis and cleavage of its components by matrix metalloproteinases. On the other hand, little is known about the complex relationship between E2, regulation of mRNA stability and deposition of ECM molecules in the uterine tissues. Therefore, the present work aimed to unveil the effects of E2 on versican mRNA levels and stability, and the deposition of the proteoglycan in the uterine ECM of ovariectomized hormone-treated mice, in a dose-related and time-dependent manner.

## Materials and methods

### Animals and tissue collection

Swiss female mice, aged 3–5 months, were used in this study. Animals were housed in a 12-h light: 12-h dark, temperature-controlled (22 °C) environment, with free access to food and water. For tissue collection, animals were anesthetized with an intraperitoneal injection of tribromoethanol (Avertin^®^) (0,025 mL/g body weight). The uteri were subsequently removed, cut with razor blades and immediately immersed in a fixative solution or stored in RNAlater solution (Sigma-Aldrich, St. Louis, MO, USA, #077K1749). National guidelines for laboratory animal care were followed, and all experiments were approved by the Institute of Biomedical Sciences Animal Ethics Committee (authorization number, 144/2002).

### Hormone replacement protocol

All females were ovariectomized (OVX), under Avertin^®^ anesthesia, twenty days before treatments. Five experimental groups were designed: 1) control mice (vehicle); 2) mice treated with 0.1 μg E2; 3) mice treated with alpha-amanitin from *Amanita phalloides* (A-amanitin) (Sigma-Aldrich, #099K4041) and 0.1 μg E2; 4) mice treated with 10 μg E2; 5) mice treated with A-amanitin and 10 μg E2. A-amanitin is a well known transcription blocker and in vivo experiments using this reagent have been previously published (Lee and Ling 2003). Briefly, mice in groups 2 and 4 received an i.p. A-amanitin injection (diluted in distilled water, 50 μg/100 g body weight). After 1 h, the animals were treated with vehicle or the respective dose of E2; 17β-estradiol (E2) (Sigma-Aldrich, #E8875) was diluted in 50 % ethyl alcohol and injected via caudal vein in a 50 μl volume.

All four treated groups, except the control group, were each divided into six subgroups, in which the animals were sacrificed after a specific period of time following hormone administration: 30 min, 1, 3, 6, 12 and 24 h (n = 5 per group).

### mRNA extraction and RT-PCR

Upon use, uterine samples, excised with sterilized razor blades and stored in RNAlater solution at −20 °C, were transferred to sterile microfuge tubes containing ceramic beads and 500 μl of Trizol reagent (Invitrogen, Calbard, CA, USA, #15596018). Samples were homogenized and total RNA extracted using the Precellys 24 homogenizer (Bertin Technologies, Saint-Quentin-en-Yvelines, France), following the manufacturer’s instructions. RNA quantity and quality (A_260_/A_280_) were assessed with a NanoDrop spectrophotometer (Thermo Fisher Scientific Inc., USA) and 1.2 % agarose gel electrophoresis. All samples were pre-treated with DNAse I for 15 min (Invitrogen, #18068-015) to remove undesired genomic DNA contamination. Reverse transcription (RT-PCR) to synthesize first-strand cDNA was performed for 1 h at 42 °C with AffinityScript QPCR cDNA Synthesis kit (PCR buffer, random primers and reverse transcriptase) (Stratagene, Cedar Creek, TX, USA, #600559) and 1 μg of total RNA, in a 20 μl reaction volume. The cDNA was diluted 1:5 for use in real-time qPCR and stored at −20 °C.

### Quantitative real-time PCR (qPCR)

Different reference genes were tested and GAPDH (Glyceraldehyde 3-phosphate dehydrogenase) was chosen as internal control for showing the most uniform expression across groups in the qPCR amplification experiments, according to the NormFinder^®^ algorithm (Andersen et al. 2004). Efficiency of each pair of primers was determined by amplification reaction with serial dilutions of a control sample (investigated range from 2.5 ng to 40 ng), which display the slope and the value of a in y = ax + b (Efficiency = 10^−1/a^). The relative levels of mRNA of the tested genes were estimated by fluorescence quantified with the RotorGene 6000 Series (Corbett Research, Australia). Reactions were performed in a total volume of 25 μl containing sterile water, 20 ng of cDNA and 900 nM of forward and reverse primers in a reaction buffer containing SYBR Green PCR master mix (Stratagene, Cedar Creek, TX, USA, #600548). The reaction conditions consisted of one step at 95 °C for 10 min, followed by 40 cycles of two steps: 30 s at 95 °C (denaturation) and 60 s at 60 °C (annealing). The melting curves obtained, which showed a single amplicon and no primer dimers, were programmed as follows: 60–95 °C with a heating rate of 0.1 °C per second and a continuous fluorescence measurement. All Cq (quantification cycle) values of the target gene (Vcn) were normalized to the expression of the reference gene (GAPDH) and the results were expressed as fold-induction relative to the expression of the calibrator sample (control vehicle), arbitrarily set to 1, using the comparative Ct method. Primers were designed with NCBI’s primer designing tool to span an exon–exon junction, in order to limit amplification only to mRNA. Primer sequences used were: Versican Forward– ^9532^TCCTGATTGGCATTAGTGAAG^9552^ and Reverse—^9689^CTGGTCTCCGCTGTATCC^9672^ (158 bp, #NM_001081249); GAPDH Forward—^840^TCTGAGGGCCCACTGAAG^857^ and Reverse– ^1060^AGGGTTTCTTACTCCTTGGAGG^1039^ (221 bp, #NM_008084.2).

### Evaluation of versican mRNA poly(A) tail length

Versican transcript poly(A) tail length was examined by a rapid amplification of cDNA ends-poly(A) test (RACE-PAT) (Sallés et al. 1999).

In brief, 200 ng of an oligo(dT) anchor (5′-GCGAGCTCCGCGGCCGCG-T12) were added to 1 μg of total RNA in a sterile RNase-free microfuge tube. The samples were denatured at 70 °C for 5 min, transferred to 42 °C and reverse transcribed for 60 min, using the AffinityScript QPCR cDNA Synthesis kit (Stratagene).

Three microliters of the RT reaction product were mixed to a reaction buffer containing MgCl_2_ (25 mM), 10 mM of each dNTP, 25 pmol of each primer (Versican, 5′-GTACTAGAGCACAGAAAATC and anchor, 5′-GCGAGCTCCGCGGCCGCG-T12), 1.25 units of platinum Taq DNA polymerase (Invitrogen Life Technologies, #10966-030) and 10x Taq DNA polymerase buffer. The PCR reaction was performed for 40 cycles; each cycle consisted of 95 °C for 30 s (denaturation), 64 °C for 1 min (annealing), and 72 °C for 1 min (extension), followed by a single final 7-min elongation step at 72 °C.

The PCR-amplified products were submitted to electrophoresis in 2.5 % agarose gel containing ethidium bromide. Amplicon sizes were estimated by densitometry and compared with a 100-bp DNA ladder (Invitrogen Life Technologies, #15628-019) using the ImageQuant TL v2005 software (Amershan Biosciences, Buckinghamshire, UK). The top of the smear indicated the longest amplified fragment, which represents the poly(A) tail size plus 212 bases upstream, according to the versican primer used.

### Light microscopy processing

Samples were fixed at 4 °C for 3 h in Methacarn (absolute methanol, chloroform and glacial acetic acid; 6:3:1), rinsed with absolute ethanol, and embedded in Paraplast (Merck, Oxford, St. Louis, MO, USA, #1.11609) at 60 °C. Samples were cut into 5 μm sections, adhered to glass slides using 0.1 % Poly-l-lysine (Sigma, St. Louis, MO, USA, #P8920), then dried at 37 °C and stored at room temperature.

### Immunoperoxidase procedure

Immunostaining was performed according to a previously established protocol (Salgado et al. 2009b). Sections were deparaffinized, hydrated and treated with 3 % (v/v) H_2_O_2_ in PBS (30 min) to block endogenous peroxidase activity. They were subsequently incubated with Chondroitinase ABC from *proteus vulgaris* (Seikagaku, Tokyo, Japan, #100330), diluted in 20 mM Tris–HCl buffer pH 6.0 (1 h at 37 °C), prior to the incubation with the primary antibody, in order to digest the glycosaminoglycan side chains and expose the epitopes to the antibody. Nonspecific staining was blocked by incubating the sections (1 h) with normal goat serum, diluted 1:1 (v/v) in PBS—10 % BSA (w/v) (room temperature). The primary antibody was a versican polyclonal antibody produced in rabbit (Lifespan, Seattle, Washington, USA, #LS-C25140). The antibody was diluted 1:50 in PBS—0.3 % (v/v) Tween 20 and incubated overnight (4 °C). The sections were then incubated with peroxidase-conjugated secondary anti-rabbit IgG (1:200) (KPL, Gaithersburg, Maryland, USA, #04-15-06), diluted in PBS (v/v) (1h30 min, at room temperature). The peroxidase reaction was visualized using 0.03 % (w/v) 3,3′-diaminobenzidine (DAB) (Sigma-Aldrich, #868272-85-9) in PBS with 0.03 % (v/v) H_2_O_2_. The sections were counterstained with Mayer’s haematoxilin. Negative control reactions consisted of replacing the primary antibodies with the respective non-immune serum at similar concentrations or by omitting the primary antibody step from the protocol. Uterine cross sections in diestrus phase of the estrous cycle were used as positive control.

A Nikon Eclipse E600 microscope was used for examining sections. Images were captured using a digital camera (Cool SNAP-Procf color; Roper Scientific, Trenton, NJ, USA) and Image Pro Plus software (Media Cybernetics, Silver Spring, MD, USA).

### Statistical analysis

Comparisons between two distinct groups (control vs treated) were analyzed by the Student t test. Multiple comparisons were performed by one-way analysis of variance (ANOVA) followed by the Student–Newman–Keuls post-test to determine significant differences among all groups, using Prism 5.0 (GraphPad Software, San Diego, CA, USA). All results were expressed as the mean ± standard error of the mean (SEM). Values of p less than or equal to 0.05 were considered statistically significant.

## Results

### Effect of E2 on versican gene expression in a dose-related and time-dependent manner

**Fig. 1:**
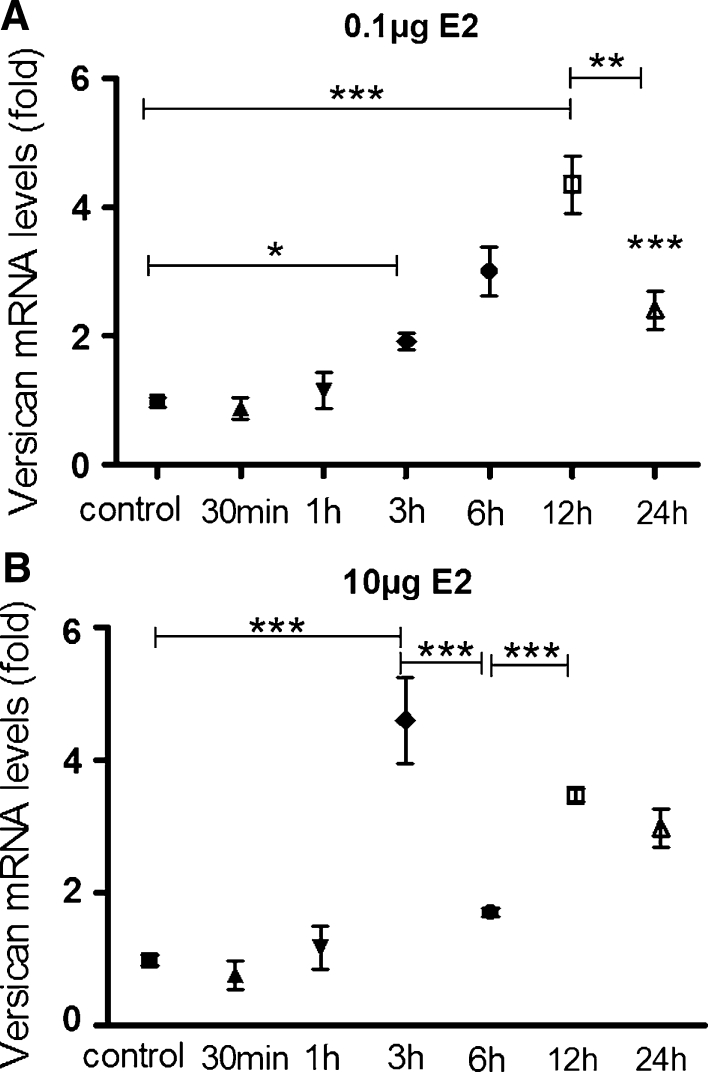
Versican mRNA expression shows significant increase from 3 h onward after administration of **a** 0.1 μg E2, when compared to the control group, reaching its peak 12 h after treatment; **b** 10 μg E2—however, after administration of the higher dose, transcript content reaches its peak within 3 h of treatment, earlier than observed in the lower E2 dose (12 h). 6 h after treatment versican transcript content significantly decreases (3 vs 6 h), increasing again 12 h after E2 administration. Multiple comparisons performed by one-way analysis of variance (ANOVA) followed by the Student–Newman–Keuls post hoc test. Values represent the mean ± SEM on five independent tissue preparations. *p < 0.05; **p < 0.01; ***p < 0.001

Versican mRNA expression showed significant increase from 3 h after administration of 0.1 μg E2 (*p* < 0.05) onward, when compared to the control group, reaching its peak 12 h after treatment (*p* < 0.001). Although there was a significant decrease in versican mRNA in the 24 h group, when compared to 12 h group (*p* < 0.001—12 vs 24 h), the content in the 24 h group was higher than in the control group (*p* < 0.001) (Fig. 1a).

Similarly to that observed in the 0.1 μg E2 group, in the group treated with 10 μg of E2, versican mRNA expression was significantly augmented only from 3 h (*p* < 0.001) onward. However, after administration of the higher dose, transcript content reached its peak within 3 h of treatment, earlier than was observed in the lower E2 dose (12 h) treated animals. Interestingly, 6 h after treatment versican transcript content significantly decreased (*p* < 0.001—3 vs 6 h), increasing again 12 h after E2 administration (Fig. 1b).

### Effect of A-amanitin on versican gene transcription arrest

**Fig. 2:**
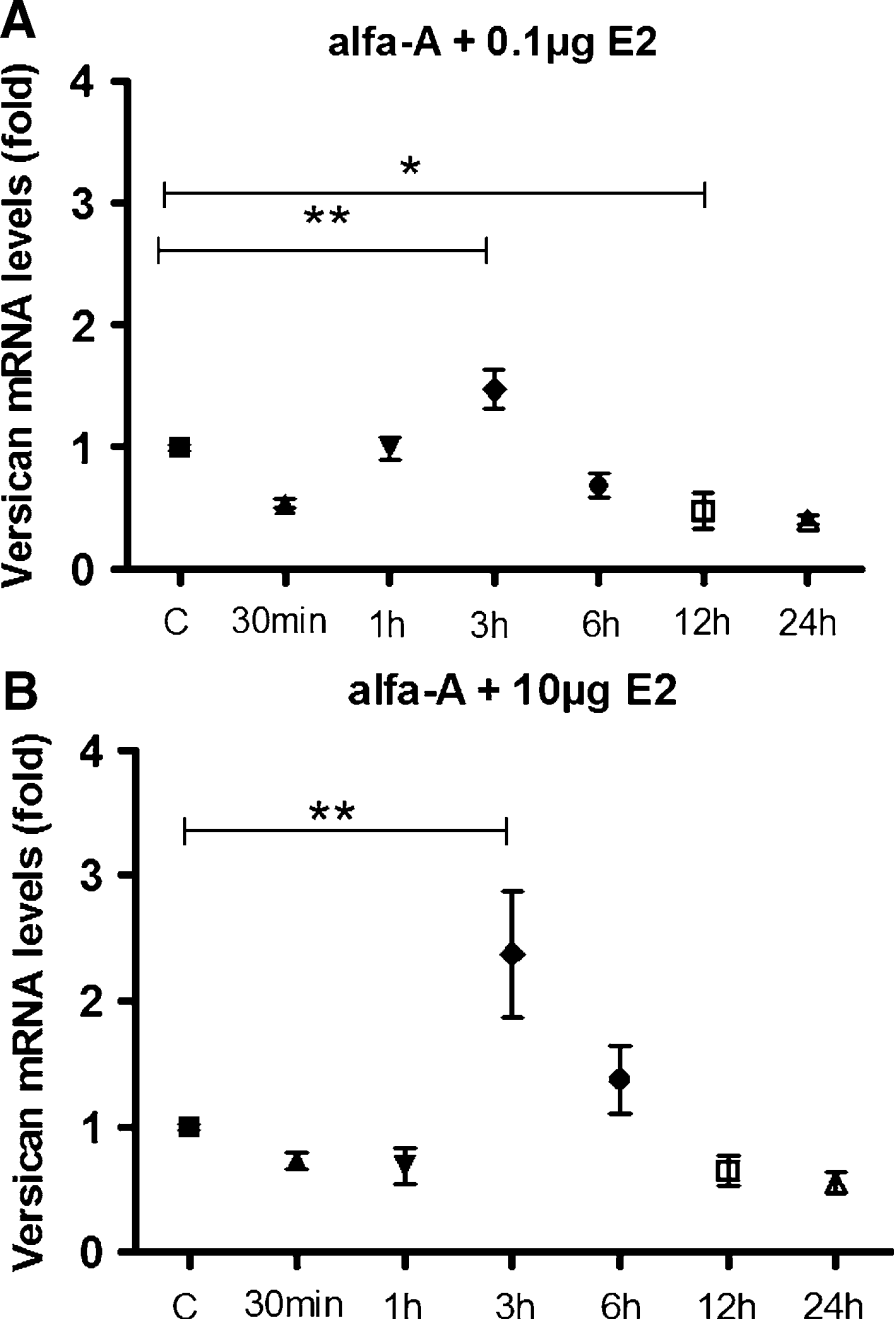
**a** and **b** Pre-treatment with A-amanitin promotes significant increase of versican mRNA 3 h after E2 administration, strongly suggesting an increase in mRNAm stability. Otherwise, 12 h after 0.1 μg E2 administration there is a significant decrease in versican mRNA levels (3 vs 12 h), and 12 h after 10 μg E2 injection versican transcript content remains unchanged, when compared to the control group, indicating that mRNA stability is no longer enhanced. The decaying curve observed shows that transcription blocking was effective. Multiple comparisons performed by one-way analysis of variance (ANOVA) followed by the Student–Newman–Keuls post hoc test. Values represent the mean ± SEM on five independent tissue preparations. *p < 0.05; **p < 0.01

Pre-treatment with A-amanitin promoted significant increase of versican mRNA 3 h after E2 administration (*p* < 0.01), strongly suggesting an increase in mRNAm stability. Otherwise, 12 h after 0.1 μg E2 administration, there was a significant decrease (*p* < 0.05) in versican mRNA levels and 12 h after 10 μg E2 injection versican transcript content remain unchanged, when compared to the control group, indicating that mRNA stability is no longer enhanced. Therefore, this decaying curve showed that transcription blocking was effective (Fig. 2a, b).

### Effect of E2 on versican transcript poly(A) tail length

**Fig. 3:**
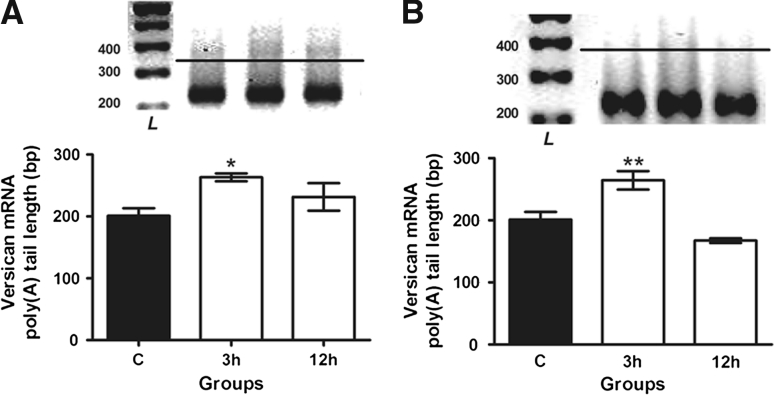
Versican mRNA poly(A) tail length analyzed by rapid amplification of cDNA ends for poly(A) test (RACE-PAT). Swiss female mice were treated for 3 and 12 h with 0.1 μg E2 (**a**) or 10 μg E2 (**b**). *Upper panels*: Smearing pattern of PCR products of C and E2-treated mice, in ethidium bromide-stained 2.5 % agarose gel. The 100 bp DNA ladder (L) was the reference for the analysis of poly(A) tail length in base pairs (bp). *Lower panels*: The maximal sizes of the amplicons, corresponding to the top of the smear of control (*black bar*), estradiol-treated mice (*white bars*) are shown in base pairs (bp). Results are expressed as mean ± SEM of 2 independent experiments (n = 4). *P < 0.05, **P < 0.01 versus Control (one-way analysis of variance with Student–Newman–Keuls as post hoc test)

In order to confirm whether E2 induces posttranscriptional modifications in versican transcript, its poly(A) tail length was analyzed. It was verified that the length of versican mRNA poly(A) tail was significantly increased 3 h after administration of 0.1 μg E2 (*p* = 0.0147) or 10 μg E2 (*p* = 0.026), when compared to the control group. No significant alteration in versican transcript poly(A) tail was observed 12 h after 0.1 μg E2 or 10 μg E2, as expected from the qPCR results obtained after transcription arrest (Fig. 3a, b).

### Analysis of versican deposition and distribution in the uterine tissues

#### Hormone treatment—0.1 μg E2

**Fig. 4:**
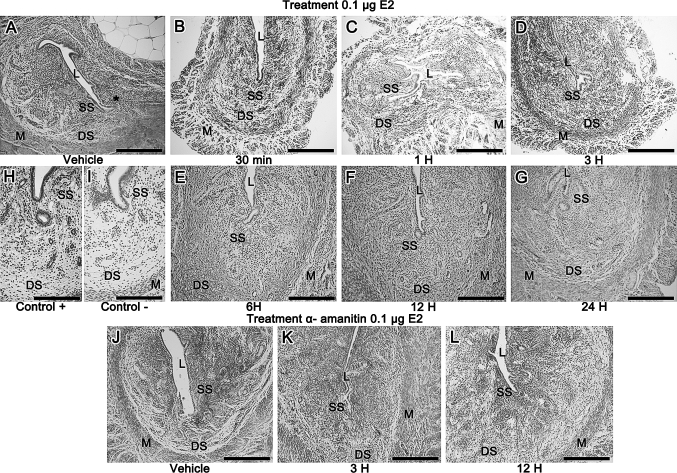
**a** In the control group (vehicle) a faint immunoreaction is observed in the subepithelial region of the antimesometrial stroma (*asterisk*); **b** 30 min; **c** 1 h and **d** 3 h after E2 injection, immunoreaction is similar to the control group; **e** 6 h; **f** 12 h and **g** 24 h after treatment there is a strong immunoreaction in the whole endometrial stroma and in the connective tissue between muscle layers; **h** and **i** show the* positive *and* negative* control, respectively. After a-amanitin treatment, immunoreaction for versican is present only in the superficial stroma in **j** control group. In **k** 3 h after treatment, a moderate immunoreaction is observed in the whole endometrial stroma; **l** In the 12 h group, the staining is restricted to the superficial stroma. *L* uterine lumen, *SS* Superficial Stroma, *DS* Deep Stroma, *M* Myometrium. *Scale Bar* = 200 μm

In the control group (vehicle) a faint immunoreaction was restricted to the subepithelial region of the antimesometrial stroma. Thirty minutes, 1 and 3 h after E2 injection, immunoreaction was similar to the control group. From 6 h onward there was a conspicuous immunoreaction in the whole stroma and in the connective tissue between muscle layers. The smooth muscle tissue of the myometrium showed no immunoreaction. Figure 4a–i show the immunohistochemistry reactions, including negative and positive control.

#### Hormone treatment—10 μg E2

**Fig. 5:**
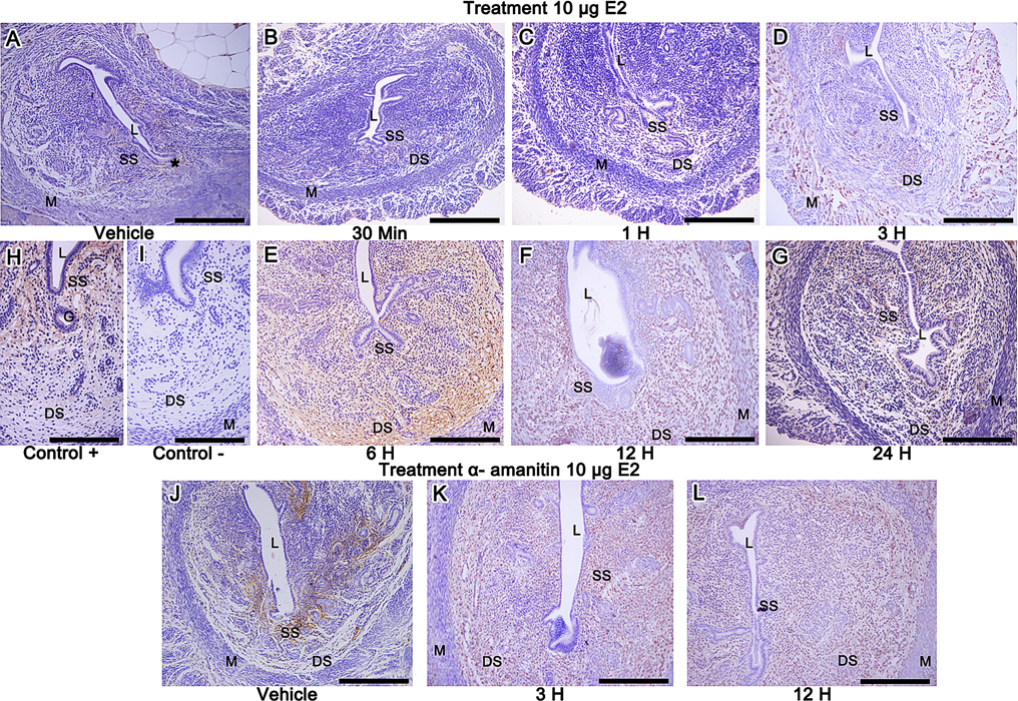
**a** In the control group (vehicle) a faint immunoreaction is observed in the subepithelial region of the antimesometrial stroma (*asterisk*); **b** 30 min and **c** 1 h after E2 injection, immunoreaction has a similar pattern of deposition than control; **d** 3 h; **e** 6 h; **f** 12 h after treatment the immunoreaction is present in the whole endometrial stroma and in the connective tissue between muscle layers, however it is weaker than in the previous dose; **g** In the 24 h group, the immunodeposition recedes; **h** and **i** show the* positive *and* negative* control, respectively. After a-amanitin treatment, immunoreaction for versican is present only in the superficial stroma in **j** control group. Interestingly, **k** 3 h after treatment a strong immunoreaction is observed in the whole endometrial stroma; L) In the 12 h group, the staining is faint from the uterine tissues. *L* uterine lumen, *SS* Superficial Stroma, *DS* Deep Stroma, *M* Myometrium, *G* endometrial gland. *Scale Bar* = 200 μm

The administration of 10 μg E2 showed that the immunoreactivity to versican in the uterine tissues was also apparent only 3 h after hormone administration. Similarly to that observed after 0.1 μg E2 injection, the immunoreaction was present in the whole endometrial stroma in the 6 and 12 h groups, however weaker than that observed in the 0.1 μg E2 groups. In addition, in the 12 h group it appears as a dot-like staining throughout the whole stroma and myometrial layers. After 24 h of E2 injection, the immunodeposition of versican recedes in the endometrial stroma (Fig. 5a–i).

#### A-amanitin and hormone treatment—0.1 μg E2

In the control group (vehicle) treated with a-amanitin, immunoreaction for versican was present only in the superficial stroma (Fig. 4j). Interestingly, 3 h after treatment a moderate immunoreaction was observed in the whole endometrial stroma (Fig. 4k), whereas it was restricted to the subepithelial region 12 h after treatment (Fig. 4l).

#### A-amanitin and hormone treatment—10 μg E2

The immunoreaction in the control group (vehicle) treated with a-amanitin is observed in Fig. 5j. After transcription block, a strong immunoreaction was observed in the whole endometrial stroma and connective tissue between muscle layers in the 3 h group. However, differently than that observed in the lower dose group, immunoreaction was faint in the uterine tissues in the 12 h group (Fig. 5k, l).

## Discussion

Hormone actions on target cells involve complex molecular mechanisms, especially in the uterus, an organ characterized by a remarkable remodeling, which occurs through synthesis, deposition and degradation of ECM molecules (Abrahamsohn and Zorn 1993).

An important variable in the context of hormone actions is the regulation of mRNA stability (Schauer 1991; Rao et al. 1994; Ing 2005). A pioneer study demonstrated that prolactin is capable of enhancing the expression of the casein gene in mammary gland, through the increase of its mRNA half-life and stability (Guyette et al. 1979). For instance, Saceda et al. (1998) showed that P4 increases its receptor mRNA half-life from 6 to 12 h in primary culture of endometrial fibroblasts. The present results suggest that the increase in versican mRNA beginning only 3 h after hormone treatment is due to alteration in its half-life induced by E2. In accordance, an in vivo study showed that exogenous estrogen administration is able to up-regulate ER transcription, as well as its protein concentration, in the uterus of ovariectomized ewe by stabilizing ER mRNA (Ing et al. 1996).

The cytosolic compartment of the cells contains a protein system formed by RNAses and their inhibitors the RNAsins, which control RNA turnover. It’s been reported that this system is regulated by E2, influencing the degradation/stabilization of cytoplasmic mRNAs (Rao et al. 1994). The decrease from 3 to 6 h after E2 administration in versican mRNA level may be a time-controlled consequence of transcript deadenilation (poly(A) tail shortening) and degradation by RNAses, followed by *de novo* synthesis in the endometrial stroma.

The decay of mRNAs is not a random process. It is finely controlled and is triggered by three main events: poly(A) tail shortening, premature arrest of translation and exonucleolytic digestion, which are not necessarily exclusive. Poly(A) tracts are gradually shortened in the cytoplasm in a mRNA-specific manner and this is normally a rate-determining event that triggers turnover of the body of the transcript (Jacobsen and Peltz 1996).

The posttranscriptional regulation mechanism reported in the present work occurs at the transcript polyadenylation/deadenylation level, leading to alteration in versican mRNA poly(A) tail length, which is directly correlated to transcript stability and translation efficiency (Bernstein et al. 1989; Serrano-Nascimento et al. 2010). The addition of adenine residues to the transcript’s 3′-untranslated region protects it from specific exoribonuclease and endonuclease attack, as demonstrated 3 h after E2 treatment when a longer transcript poly(A) tail was observed.

Interestingly, our results from experiments in which mice were treated with A-amanitin to block transcription showed an unexpected increase in versican mRNA levels 3 h after treatment, a phenomenon known as superinduction of mRNAs, which may occur upon treatment with transcriptional inhibitors. In fact, superinduction has been previously observed by Lee and Ling (2003), whose work proposes it could be due to the blockage of expression of factors involved in mRNA degradation, induced by a specific treatment, leading to stabilization and accumulation of mRNA pools in the cytoplasm.

The decaying curve on versican mRNA levels after transcription blocking and the analysis of its poly(A) tail length strengthen this hypothesis. Transcripts tend to be more stable with a longer poly(A) tail, thus 3 h after E2 injection versican mRNA content is posttranscriptionally modulated, whereas 12 h after treatment there is a transcriptional modulation of versican expression.

The immunohistochemical analysis of versican deposition in the uterine ECM corroborates the molecular findings. In the control group (vehicle) there is a weak reaction in the subepithelial stroma. We infer that ovariectomy doesn’t entirely suppress the stimulus on versican expression due to a basal hormonal modulation from other sources, such as the adrenal gland. It is worth mentioning that there is a clear compartmentalization of the endometrial stroma into superficial and deep regions, formed by genetically distinct cell populations (Oliveira et al. 1998; Zorn et al. 2003). The difference in the immunodeposition pattern observed between both doses is certainly due to a dose-dependent effect of E2 on versican expression. Besides mRNA degradation and therefore translation arrest, one should also account for the action of ADAMTS (A Disintegrin And Metalloproteinase with ThromboSpondin motifs), classic proteoglycan cleavers, that act as versicanases in extracellular matrices (Lee et al. 2005; Stanton et al. 2011) and are indeed produced by uterine epithelial and stromal cells under hormone regimen (unpublished data, manuscript in preparation).

In conclusion, the present results demonstrate for the first time that E2 modulates the ECM proteoglycan versican, at the transcriptional and posttranscriptional levels by regulating the rate of transcription, mRNA stability, and its protein deposition (and degradation) in the ECM in a dose-related and time-dependent manner. It is likely that similar mechanisms act in the remarkable and swift modulation observed in other ECM molecules present in the uterine tissues.
